# Comprehensive Profiling of ceRNA (circRNA-miRNA-mRNA) Networks in Hypothalamic-Pituitary-Mammary Gland Axis of Dairy Cows under Heat Stress

**DOI:** 10.3390/ijms24010888

**Published:** 2023-01-03

**Authors:** Hanfang Zeng, Haibin Xia, Xinling Wang, Yue Wang, Jian Fang, Shujie Li, Yunfei Zhai, Zhaoyu Han

**Affiliations:** College of Animal Science and Technology, Nanjing Agricultural University, Nanjing 210095, China

**Keywords:** heat stress, hypothalamic-pituitary-mammary gland axis, circRNA, ceRNA network, dairy cow

## Abstract

Heat stress (HS) is directly correlated with mammary gland dysfunction and the hypothalamic-pituitary-mammary gland (HPM) axis is involved in regulating stress responses and lactation in dairy cows. Circular RNAs (circRNAs) play major roles in regulating transcription and post-transcription but their expression in the HPM axis of dairy cows under HS is still unclear. In the present study, we performed RNA sequencing to identify diferentially expressed (DE) circRNAs, DE microRNAs(miRNAs) and DEmRNAs, and performed bioinformatics analysis on those in HPM axis-related tissues of heat-stressed and normal cows. A total of 1680, 1112 and 521 DEcircRNAs, 120, 493 and 108 DEmiRNAs, 274, 6475 and 3134 DEmRNAs were identified in the hypothalamic, pituitary, and mammary gland tissues, respectively. Gene ontology (GO) and Kyoto encyclopedia of genes and genomes (KEGG) enrichment analyses indicated that the MAPK signaling pathway is potentially a key pathway. Competitive endogenous RNA (ceRNA) networks related to HS response and lactation regulation were established in three tissues. In conclusion, our results indicate that HS induces differential circRNA expression profiles in HPM axis-related tissues, and the predicted ceRNA network provides a molecular basis for regulating the stress response and lactation regulation in heat-stressed dairy cows.

## 1. Introduction

Heat stress syndrome is a systemic indication that includes non-specific defense responses and specific disorders of the body systems when cows are stimulated by excessive temperatures that exceed their thermoregulatory ability. Heat stress (HS) affects various physiological and biochemical indexes and reduces feed intake, immunity, and reproductive performance of dairy cows [1]. Importantly, HS inhibits the proliferation of bovine mammary epithelial cells (BMECs), aggravates cellular oxidative stress, and promotes BMECs apoptosis [2] and mammary gland autophagy [3], thereby reducing lactation performance [4]. Regulation of the hypothalamic-pituitary endocrine axis is crucial in response to stimulation of the organism by the external environment [5].

The hypothalamus controls the pituitary endocrine system, and they are thought of as a structural and functional unit, regulating important physiological processes such as thermoregulation and feeding [6,7]. Prolactin (PRL) secreted by the pituitary is essential for maintaining lactation [8]. Under stress, glucocorticoid levels in the blood can rapidly increase under the influence of the hypothalamic-pituitary-adrenal cortex (HPA) axis to enhance the body’s resistance to adverse environment [9]. Excitation of hypothalamic- pituitary-thyroid (HPT) axis can regulate the oxygen consumption rate of most tissues through the thyroid hormone, altering the thermogenic effect of the body [10]. However, information about HS effects on hormonal changes related to the hypothalamic-pituitarymammary gland (HPM) axis and their regulatory mechanisms in dairy cows remains limited.

Non-coding RNAs (ncRNAs) are widespread in organisms and participate in stress responses, endocrine regulation, mammary gland development, and lactation physiology [11,12]. Circular RNAs (circRNAs), a type of ncRNAs, are covalently closed circular transcripts produced by reverse splicing, making them more stable than linear RNA [13]. Accumulating evidence suggests that circRNA can be used as a proliferation regulator of mammary epithelial cells and participate in the synthesis of milk protein and milk fat [14,15]. MicroRNA, as a highly conserved small molecule ncRNA, can regulate various physiological and pathological processes. Competitive endogenous RNAs (ceRNAs) refer to sets of RNAs that carry the same miRNA response element (MRE) and can compete for miRNA binding [16]. Studies have shown that circRNAs contain numerous miRNA binding sites and inhibit the function of target genes by adsorbing miRNA [17,18]. Despite this, few studies exist on the differential expression (DE) of circRNA and the regulatory mechanism of ceRNAs in the HPM axis in dairy cows under HS.

This study was performed to evaluate the regulation of HPM axis under HS using RNA sequencing (RNA-seq), which identified and clarified the role of ceRNAs in regulating HS in dairy cows. Our results provide a theoretical basis for the study of the molecular mechanism in the HPM axis.

## 2. Results

### 2.1. Differences in Endocrine Hormones, Antioxidant Enzymes and Heat Shock Proteins (HSPs) between NHS and HS Groups

**Figure 1:**
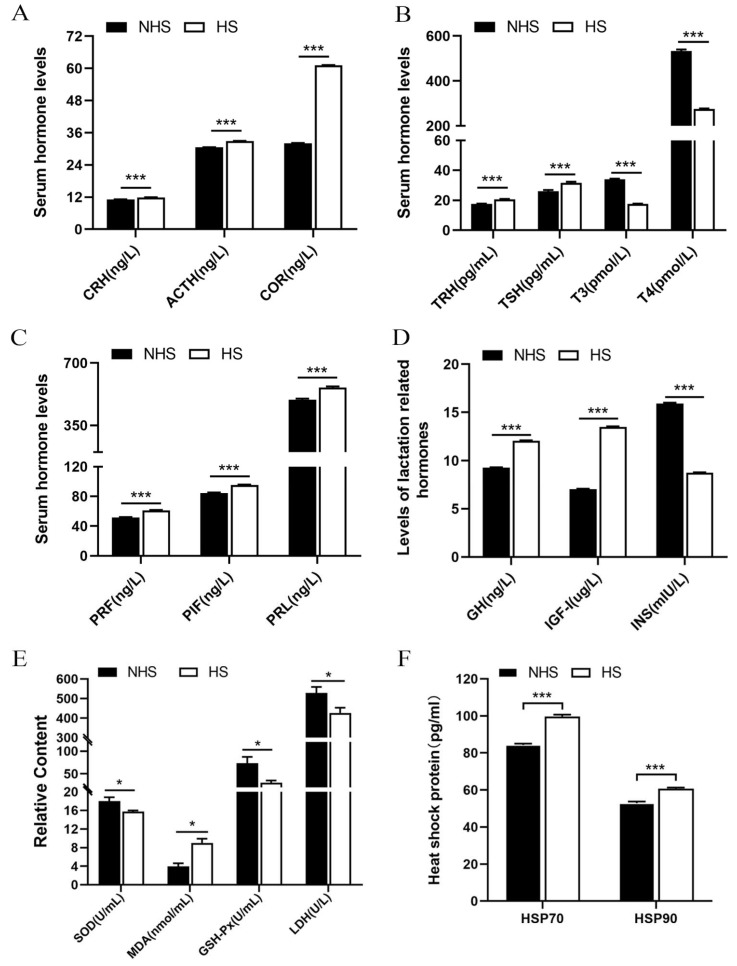
Differences in endocrine hormones, antioxidant enzymes and heat shock proteins between the NHS and HS groups. (**A**–**D**) serum endocrine hormones. (**E**) serum antioxidant enzymes. (**F**) serum heat shock protein. Data are presented as the mean ± SEM (*n* = 20 per group); * *p* < 0.05, *** *p* < 0.001.

HS significantly increased the levels of HPA axis-related hormones CRH, ACTH and COR (Figure 1A, *p* < 0.01), in the serum of dairy cows. HPT axis-related hormones TRH and TSH significantly increased (*p* < 0.01), while those of T3 and T4 significantly decreased (Figure 1B, *p* < 0.01) under HS. Compared to the NHS group, PRF, PIF and PRL significantly increased (Figure 1C, *p* < 0.01) in the HS group. Meanwhile, lactation related hormones GH and IGF-1 significantly increased (*p* < 0.01), while INS significantly decreased (*p* < 0.01) in the serum of heat-stressed dairy cows (Figure 1D). Moreover, SOD, GSH-Px and LDH activity in the serum of dairy cows significantly decreased (*p* < 0.01), while MDA content significantly increased (*p* < 0.01) under HS condition (Figure 1E). Lastly, the levels of HS marker proteins, HSP70 and HSP90, significantly increased (Figure 1F, *p* < 0.01) in the serum of heat-stressed dairy cows.

### 2.2. Histomorphological Observations of HPM-Axis Tissues and Neurotransmitters in Hypothalamus of NHS and HS Groups

**Figure 2:**
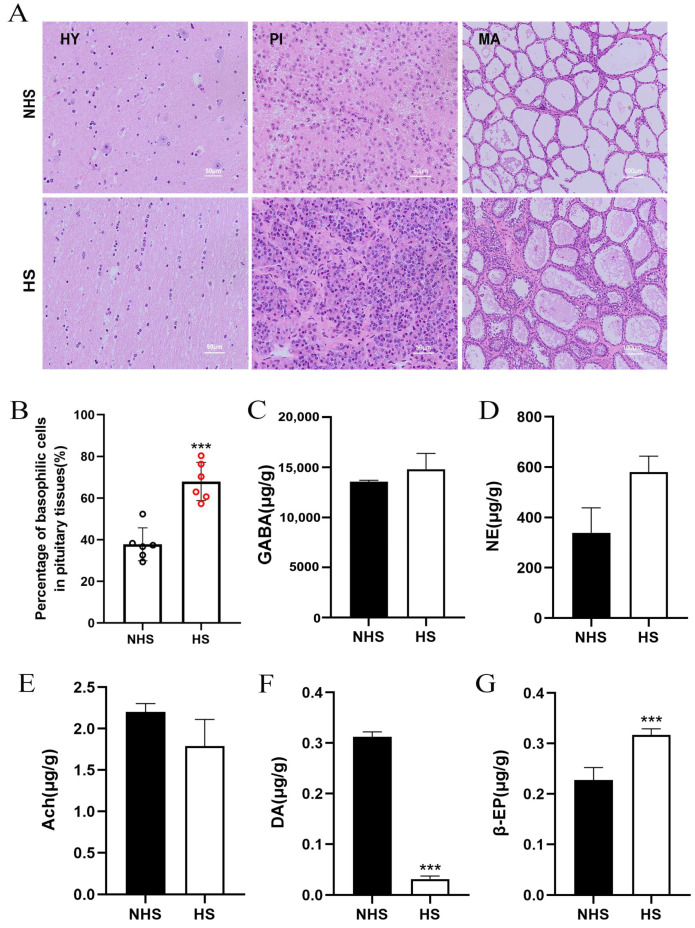
Histomorphological observation of HPM-axis related tissues and neurotransmitters in hypothalamus between the NHS and HS groups. (**A**) Histomorphological observation of HPM-axis related tissues. HY: hypothalamus; PI: pituitary; MA: mammary gland. (**B**) The percentage of basophils in pituitary. (**C**–**G**) Neurotransmitters in hypothalamus. Data are presented as the mean ± SEM (*n* = 6 per group); *** *p* < 0.001.

As shown in Figure 2A, the hypothalamic histology was not significantly different between the NHS and HS groups. However, there are histological differences between the NHS group and the HS group in the pituitary and mammary glands. Among them, the percentage of basophils in the pituitary tissue significantly increased under the HS condition (Figure 2B, *p* < 0.01). Mammary gland acini in the NHS group were round, but those in the HS group were significantly shrunken with a thicker acinar wall and their round shape was lost (Figure 2C). Subsequently, we detected neurotransmitters in the hypothalamus and found that DA significantly decreased while β-EP levels significantly increased under HS (Figure 2F,G *p* < 0.01). However, GABA, NE and Ach had no significant effect on the hypothalamic tissues of heat-stressed dairy cows (Figure 2C–E, *p* > 0.05).

### 2.3. Identification and Characterization of circRNAs in HPM Axis Tissues of Dairy Cow under Heat Stress

**Figure 3:**
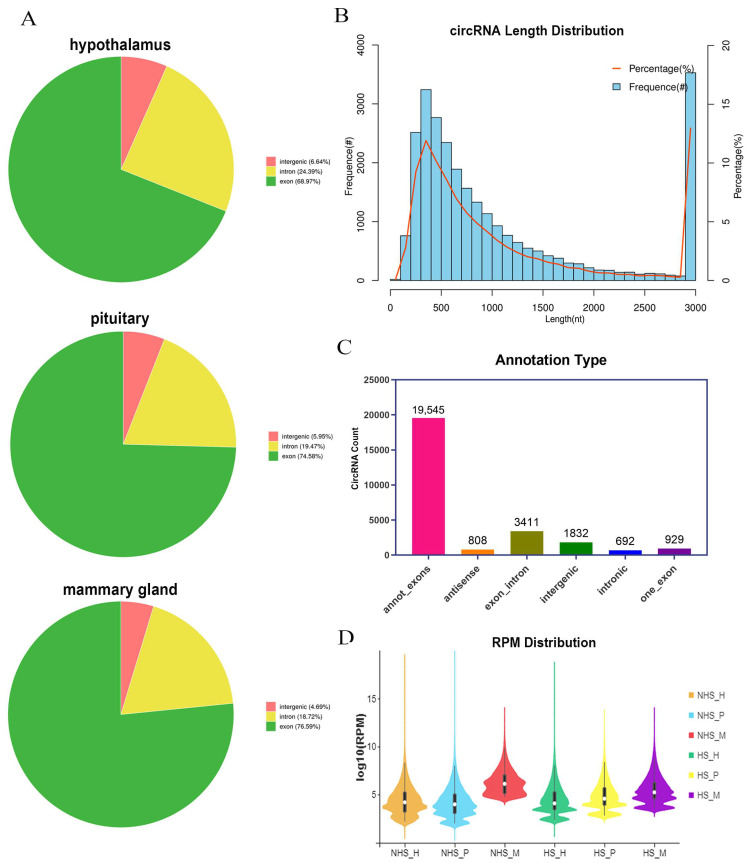
Identification and characterization of circRNAs. (**A**) Reads align region statistics. (**B**) circRNAs length distribution. (**C**) Annotation type of circRNAs. (**D**) Sample violin diagram in HPMaxis-related tissues between the NHS and HS groups.

Expression of circRNAs in the NHS and HS groups were detected and high-quality clean reads obtained to identify circRNAs (Supplementary Table S1). In the 18 sequencing libraries, 81.08%–85.63% of the reads were mapped to the reference genome (Supplementary Table S2). Approximately 68.97%, 74.58% and 76.59% of the reads in the hypothalamus, pituitary and mammary gland, respectively, were compared to exons (Figure 3A). Based on the filtering criteria, we conducted length distribution statistics and annotated circRNAs. The percentage and frequence of circRNAs with length between 300–400 nt (11.91%) and greater than 3000 nt (12.96%) are relatively high (Figure 3B), and the main annotation type was “annot_exons” (Figure 3C). A violin diagram is generally used for the visualization of gene abundance and expression and can show data density at any location. As shown in Figure 3D, the circRNA expression abundance and data density of each experimental group are appropriate. Therefore, we conducted subsequent analysis.

**Figure 4:**
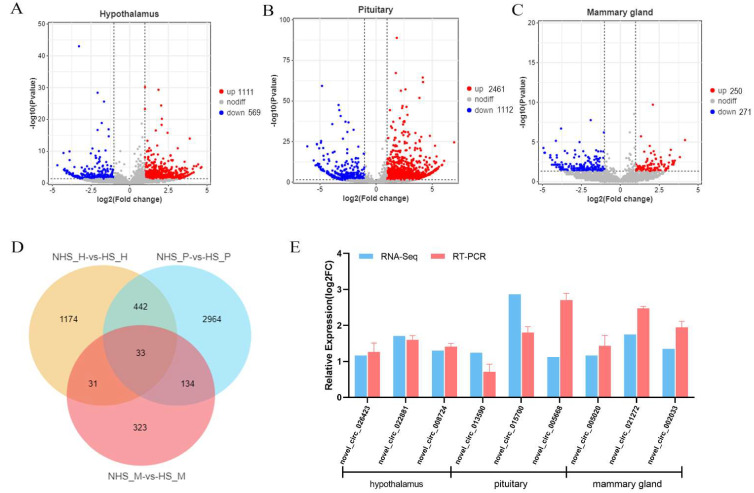
(**A**) Volcano plot of DEcircRNAs in hypothalamus. (**B**) Volcano plot of DEcircRNAs pituitary. (**C**) Volcano plot of DEcircRNAs mammary gland. Red dots represent up-regulated circRNAs and blue dots represent down-regulated circRNAs. (**D**) Venn diagram of DEcircRNAs in the HPM axis-related tissues between NHS and HS groups. (**E**) Comparison of the circRNAs expression levels determined by RNA-seq and RT-qPCR.

Furthermore, a total of 1680, 1112 and 521 DE circRNAs were identified in hypothalamus, pituitary and mammary gland, respectively. We found that 1111, 2461 and 250 circRNAs were up-regulated, and 569, 1112 and 271 circRNAs were downregulated in the three tissues (Figure 4A–C). Moreover, 33 circRNAs were shared in these tissues (Figure 4D). To verify the RNA-seq results, we randomly selected three circRNAs from HPM axis-related tissues and detected their expression using RT-qPCR. The results were highly consistent with those of RNA-seq (Figure 4E). 

### 2.4. Functional Analysis of the Source Genes of DEcircRNAs

**Figure 5:**
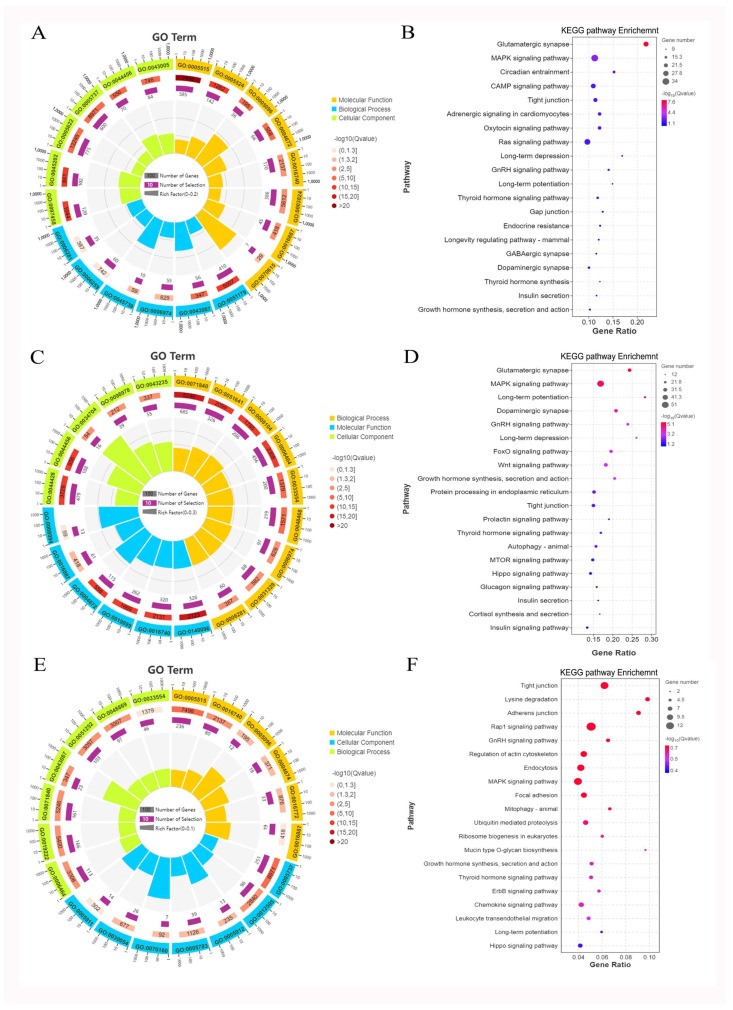
GO functional enrichment and KEGG pathway analysis on the source genes of DEcircRNAs in the hypothalamus (**A**,**B**), pituitary (**C**,**D**), and mammary gland (**E**,**F**) in dairy cows under heat stress.

To determine the biological processes associated with the source genes of the DE circRNAs in HPM axis tissues, we conducted GO and KEGG pathways enrichment analysis. In the hypothalamus, most source genes were significantly (*p* < 0.01) enriched in protein binding (GO:0005515), ATP binding (GO:0005524), regulation of GTPase activity (GO:0043087), and cellular response to DNA damage stimulus (GO:0006974) (Figure 5A). The KEGG pathways were in glutamatergic synapse, MAPK signaling pathway, cAMP signaling pathway, GABAergic synapse, thyroid hormone synthesis, and insulin secretion in the hypothalamus (Figure 5B, *p* < 0.05). In the pituitary, cellular component organization or biogenesis (GO:0071840), catalytic activity, acting on a protein (GO:0140096), and cellular protein modification process (GO:0006464) were significantly enriched (Figure 5C, *p* < 0.01). The KEGG pathways were mainly enriched in the MAPK signaling pathway, hormone synthesis, secretion, and actin, autophagy (Figure 5D, *p* < 0.05). The GO terms were enriched in cellular protein modification process (GO:0006464), transferase activity (GO:0016740), and regulation of GTPase activity (GO:0043087) in the mammary gland (Figure 5E, *p* < 0.01). Additionally, more source genes were enriched in tight junction, MAPK signaling pathway, and hormone synthesis and secretion pathway (Figure 5F, *p* < 0.05).

### 2.5. Identification of DE miRNAs in HPM Axis Tissues of Dairy Cows under Heat Stress

**Figure 6:**
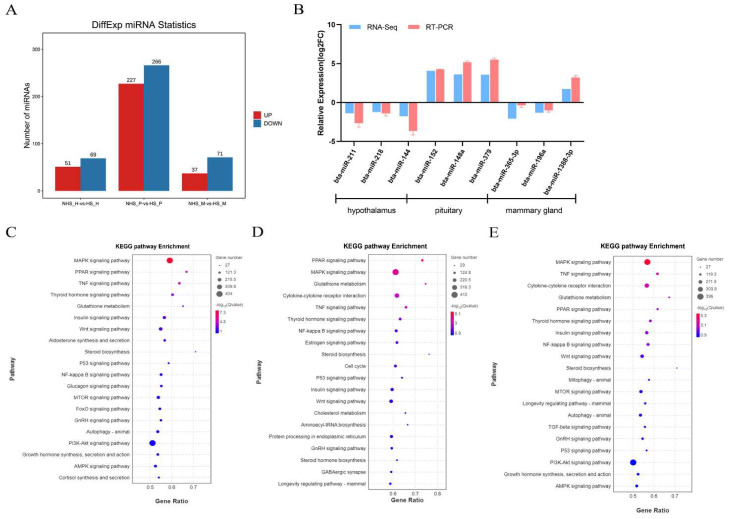
(**A**) Statistics of differential expression of miRNAs. (**B**) Comparison of the miRNAs expression levels determined by RNA-seq and RT-qPCR. (**C**–**F**) KEGG pathway analysis on the target genes of DE miRNAs in the hypothalamus, pituitary and mammary gland in dairy cows under heat stress.

To investigate the miRNA expression profiles in the HPM axis tissues of dairy cows under HS, 18 small RNA libraries were constructed and sequenced. The results identified 120, 493 and 108 DEmiRNAs in the hypothalamus, pituitary gland, and mammary gland, respectively, of which 51, 227, and 37 miRNAs were upregulated and 69, 266, and 71 miRNAs were downregulated (Figure 6A). Furthermore, we verified the expression of miRNAs in the hypothalamus (bta-miR-211, bta-miR-218, and bta-miR-144), pituitary gland (bta-miR-152, bta-miR-148a, and bta-miR-379), and mammary gland (bta-miR-365-3p, bta-miR-196a, and bta-miR-1388-3p) using RT-qPCR. These results were highly consistent with those of RNA-seq (Figure 6B). Subsequently, we analyzed the function of target genes of DEmiRNAs. As shown in Supplementary Figure S1, 2499, 2649, and 2454 GO terms were enriched in the hypothalamus, pituitary, and mammary gland, respectively. Most target genes of DEmiRNAs were significantly (*p* < 0.05) enriched in the MAPK signaling pathway and hormone synthesis and secretion in the HPM-axis (Figure 6C–E).

### 2.6. Identification of DEmRNAs in HPM Axis Tissues of Dairy Cows under Heat Stress

**Figure 7:**
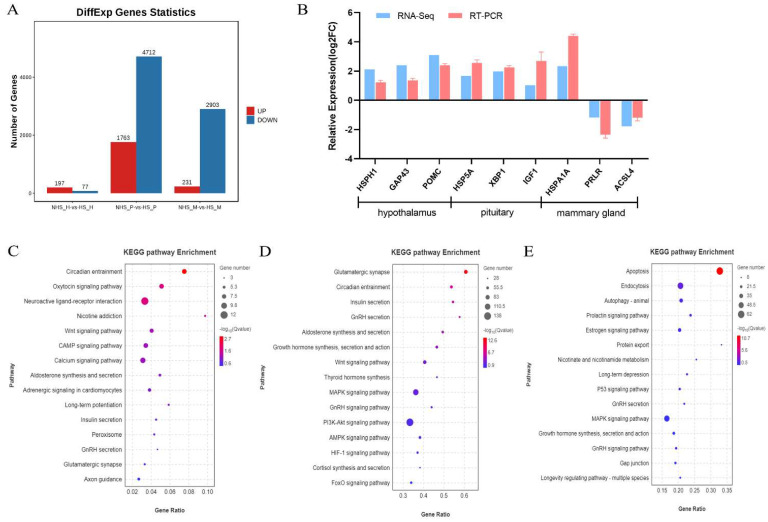
(**A**) Statistics of differential expression of mRNAs. (**B**) Comparison of the mRNAs expression levels determined by RNA-seq and RT-qPCR. (**C**–**F**) KEGG pathway analysis on the DE mRNAs in the hypothalamus, pituitary and mammary gland in dairy cows under heat stress.

The analysis identified 197, 1763, and 231 upregulated DEmRNAs, and 77, 4712, and 2903 downregulated DEmRNAs in the hypothalamus, pituitary, and mammary gland, respectively (Figure 7A). We verified the expression of randomly selected mRNAs (*HSPH1*, *GAP43*, *POMC*, *HSP5A*, *XBP1*, *IGF1*, *HSPA1A*, *PRLR*, and *ACSL4*) from HPM axis-related tissues by RT-qPCR. The results were all consistent with those obtained using RNA-seq (Figure 7B). For the DEmRNAs, 341, 1292, and 756 GO terms (*p* < 0.01) were significantly enriched in the hypothalamus, pituitary gland, and mammary gland, respectively (Supplementary Figure S2). Among the significantly enriched KEGG pathways, circadian entrainment was the most significantly enriched in the hypothalamus (Figure 7C, *p* < 0.05), and the cAMP signaling pathway in the pituitary (Figure 7D, *p* < 0.05). Apoptosis, autophagy, prolaction signaling pathway, and MAPK signaling pathway were significantly enriched in the mammary gland using KEGG analysis (Figure 7E, *p* < 0.05).

In addition, we focused on genes related to *HSPs*, hormones in HPM axis, and mammary gland apoptosis to explore the possible effects of HS on the physiological processes of dairy cows (Supplementary Table S3). We found that most of the genes were encoded *Hsp40*, *Hsp70*, *Hsp90*, and *Hsp110*. Among these, *HSPH1*, *HSF2BP*, *HSPA5*, *HSPA2*, *HSPA1A*, and *DNAJB1* were significantly upregulated under HS (*p* < 0.01), while other genes were significantly downregulated (*p* < 0.01). In the thermal environment, the expression of thyroid stimulating hormone subunit beta (*TSHB*), growth hormone releasing hormone receptor (*GHRHR*), and thyroid hormone receptor beta (*THRB*) were significantly up regulated (*p* < 0.01) in the hypothalamus of dairy cows. The expression of growth hormone 1 (*GH1*), follicle stimulating hormone subunit beta (*FSHB*), *GHRHR*, growth hormone secretagogue receptor (*GHSR*), and thyroid stimulating hormone receptor (*TSHR*) were significantly up regulated (*p* < 0.01) in the pituitary gland, while *TRH* and corticotropin releasing hormone binding protein (*CRHBP*) were significantly down regulated (*p* < 0.01) under HS. Lastly, HS led to the significant upregulation (*p* < 0.01) of the expression of *PRL*, *GH1* and *FSHB* in the mammary gland, while *PRLR*, *GHR*, *IGF1*, and insulin-like growth factor-binding protein 1 (*IGFBP1*) were significantly downregulated (*p* < 0.01). Surprisingly, DE mRNAs associated with apoptosis are significantly downregulated (*p* < 0.01) in the mammary gland, including the BCL2 apoptosis regulator (*BCL2*), apoptosis inducing factor mitochondria associated 2 (*AIFM2*) and DNA damage induced apoptosis suppressor (*DDIAS*) (Supplementary Table S3).

### 2.7. Regulatory ceRNA Network (circRNA-miRNA-mRNA) in the HPM Axis of Dairy Cows under Heat Stress

A ceRNA network was constructed to clarify the relationship among DEcircRNAs, DEmiRNAs and DEmRNAs in the HPM axis of dairy cows under HS.

**Figure 8:**
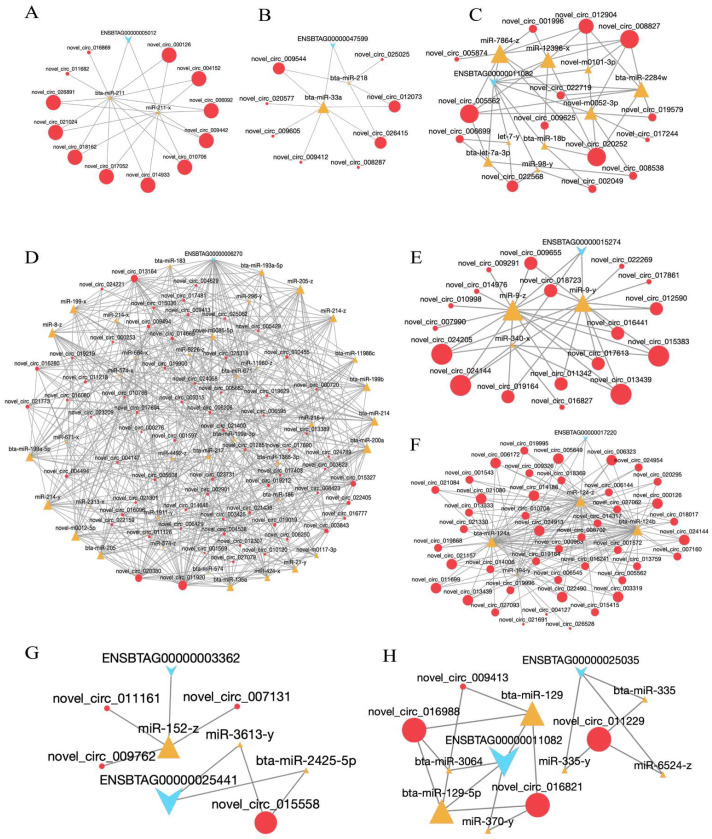
ceRNA regulatory networks in HPM axis of dairy cows under heat stress. (**A**,**B**) Subnetwork of *HSPH1* and *GHRHR* in hypothalamus. (**C**–**F**) Subnetwork of *IGF1*, *HSP90AA1*, *PRL*, and *GH1* in pituitary. (**G**,**H**) Subnetwork of *HSP90B1*, *HSPA1A*, *PRLR* and *IGF-1* in mammary gland.

A tatal of 283 circRNAs, 75 miRNAs, and 124 mRNAs formed 895 circRNA-mRNA pairs in the hypothalamus (Supplementary Table S4). The ceRNA network about HSP family H (Hsp110) member 1 (*HSPH1*, ENSBTAG00000005012) is shown in Figure 8A. *HSPH1* expression was regulated by 12 circRNAs and two miRNAs and contained 22 circRNA-mRNA pairs. Among these, novel_ circ_ 000126 and bta-miR-211 were the main regulatory factors. The expression of *GHRHR* (ENSBTAG00000047599) was regulated by eight circRNAs and two miRNAs (bta-miR-33a and bta-miR-218), and novel_circ_002325 was the main regulatory factor (Figure 8B). 

In the pituitary, the ceRNA regulatory network contained 149,018 circRNA-mRNA pairs, and included 2782 circRNAs, 490 miRNAs, and 4242 mRNAs (Supplementary Table S5). The expression of HSP 90 alpha family class A member 1 (*HSP90AA1*, ENSBTAG00000006270) was regulated by 66 circRNAs and 37 miRNAs, and novel_circ_011920 was the main regulatory factor (Figure 8D). Moreover, *IGF1*(ENSBTAG00000011082, Figure 8C), *PRL* (ENSBTAG00000015274, Figure 8E), and *GH1*(ENSBTAG00000017220, Figure 8F) expressions were regulated by 14, 18, and 44 circRNAs, and nine, three and four miRNAs, respectively. The most significant differences were found in novel_circ_005562 and miR-7864-z that regulated *IGF1*, novel_circ_013439 and miR-9-z/miR-9-y that regulated *PRL*, and novel_circ_006323 and bta-miR-124a that regulated *GH1*.

In the mammary gland, the ceRNA regulatory network contained 2268 circRNA- mRNA pairs, and included 145 circRNAs, 82 miRNAs, and 922 mRNAs (Supplementary Table S6). We found that novel_ circ_ 007131, novel_ circ_ 009762 and novel_ circ_ 011161 co-regulated miR-152-z, and affected the expression of the target gene *HSP90B1* (ENSBTAG00000003362, Figure 8G). Moreover, the expression of *HSPA1A* (ENSBTAG00000025441, Figure 8G) was regulated by novel_circ_015558, and bta-miR-2425-5p and miR-3613-y. The subnetworks of novel_circ_011229- *PRLR* (ENSBTAG00000025035) are shown in Figure 8H. *PRLR* was regulated by bta-miR-335, miR-335-y, and miR-6524-z. *IGF-1* (ENSBTAG00000011082) expression was regulated by three circRNAs (novel_circ_009413, novel_circ_016821, and novel_circ_016988) and four miRNAs (bta-miR-129, bta-miR-129-5p, miR-370-y, and bta-miR-3064). Of these, novel_circ_016821 and bta-miR-129 were the main regulatory factors (Figure 8H).

## 3. Discussion

The decline in lactation performance of dairy cows caused by HS should not be underestimated. The hypothalamic-pituitary-endocrine axis is crucial for the stress response and lactation, and the expression of related pathways and key genes to relieve HS is also important information. Consequently, in the present study, we determined the endocrine hormones of the HPM axis responsible, and established a hypothalamic, pituitary, and mammary gland ceRNA (circRNA-miRNA-mRNA) network using RNA-seq data from dairy cows under HS.

Under high temperatures, cows attempt to promote heat dissipation or reduce metabolic heat production to maintain physiological homeostasis. Excessive accumulation of reactive oxygen species (ROS) in cells caused by HS induces antioxidant dysfunction [19]. The scavenging capacity of ROS is mainly reflected in the activity of antioxidant enzymes [20]. In the present study, MDA levels in the serum of dairy cows increased significantly, while that of SOD, GSH-Px and LDH decreased significantly. Therefore, our results confirm that dairy cows experience oxidative stress under HS.

Hormones are highly effective bioactive substances secreted by the endocrine glands or scattered endocrine cells and play a regulatory role through tissue fluid or blood transmission. HS stimulates the HPA axis and promotes the secretion of ACTH, which enhances the activity of the adrenal gland and promotes the secretion of adrenaline and COR to resist the stress response [21]. The secretion of TSH from the pituitary is regulated by TRH from the hypothalamus [22]. T3 and T4 increase oxygen consumption and heat production in the body by promoting the decomposition and oxidation of sugar, fat, and protein [23,24]. The present study found that HS reduces the secretion of T3 and T4 but TSH and TRH secretion, and the expression of TRHR and TSHR significantly increased in heat-stressed cows. This is related to the negative feedback effect of T3 and T4 concentrations in the blood on the release of TSH and TRH. A variety of endocrine hormones regulate the growth, development, and lactation of the mammary gland [8]. The synthesis and secretion of PRL, a polypeptide hormone secreted by the anterior pituitary, is regulated by hypothalamic neurosecretory cells, providing PRF and PIF to the pituitary [24]. In this study, serum PRF and PIF levels in heat-stressed cows increased, which was related to the excitation of the hypothalamic-pituitary axis caused by the stress response. Interestingly, serum GH and IGF-1 levels increased significantly but the expression of GHR and IGF-1 decreased under thermal stimulation, which may affect the growth and function of BMECs. DA released from the hypothalamus binds to the D2 receptor (D2R) in the anterior pituitary, which can inhibit PRL secretion [25]. Moreover, the release of methionine enkephalin from the hypothalamus can also promote the secretion of PRL [26]. Our results showed that HS decreased the secretion of DA in the hypothalamus, but significantly increased the secretion of β-EP. Therefore, the hypothalamic–pituitary axis maintains the normal secretion of PRL in the HS environment through positive and negative feedback.

HSPs are highly conserved stress-response proteins promoting cell survival under various adverse conditions [27]. In our study, 42 DE HSPs were identified in HPM axis-related tissues by RNA-seq, and the expression levels of *HSP40*, *HSP70*, and *HSP90* families were different under HS. As molecular chaperones, HSPs are involved in protein folding, transport, and stability [28]. It is worth noting that HSPs with high molecular weight are ATP-dependent proteins [29]. Moreover, HSPs can be used as signaling molecules to inhibit apoptosis and maintain cell homeostasis after adverse stimulation [29,30]. The results of our GO analysis showed significantly enriched terms related to ATPase activation, ATP binding, and cellular response to stress. Consequently, we speculate that HSPs are important regulators in HPM axis-related tissues of heat-stressed dairy cows.

The mechanisms of ncRNAs in lactation and stress response have been studied; they play an important role in regulating RNA transcription, protein translation, and protein interaction [31,32,33]. In our study, a total of 1680, 1112, and 521 DE circRNAs were identified from the in the hypothalamic, pituitary, and mammary gland tissues, respectively. GO analysis identified biological processes related to the stress response of DEcircRNA source genes in three tissues, including cellular response to stress, regulation of cellular response to stress, stress-activated MAPK cascade, and stress-activated protein kinase signaling cascade. Subsequently, KEGG pathway enrichment analysis showed that the DEcircRNAs have roles mainly in MAPK signaling pathway, tight junction, GnRH signaling pathway, and hormone synthesis, secretion and action, which have been shown to be closely related to the stress response and physiological process of lactation. It is worth noting that the MAPK family is a group of serine/threonine kinases, which can be activated by cytokines, neurotransmitters, hormones, and cell stress. The MAPK pathways regulate a variety of cellular activities, including proliferation, differentiation, survival, and death [34]. Previous studies have shown that the MAPK signaling pathway is closely related to HSP70. MAPK can activate and up regulate the expression of heat shock factor 1 (HSF1), and promote the combination of HSF1 and HSP70 heat shock elements, thereby increasing the synthesis of HSP70 [35]. Accumulating evidence has indicated that HS stimulation induces MAPK activation, thereby affecting cell proliferation and apoptosis [36,37]. Additionally, external temperature stimulation leads to loosening of tight junctions in intestinal epithelial cells, causing gastrointestinal dysfunction [38]. A study has shown that HS affects the expression of tight junction proteins (such as ZO-1, claudin-1, and claudin-4) by regulating the ERK1/2-MAPK signaling pathway [39]. The localization of these proteins is maintained by the activation of HSPs by MAPK [38]. Therefore, the MAPK signaling pathway, as a key pathway regulating the HPM axis in heat-stressed dairy cows, should be investigated in subsequent studies.

It is widely known that circRNAs have MREs that can act as ceRNAs to regulate miRNAs and mRNAs to exert their biological functions [40]. For example, a previous study has shown that circ09863 alleviates the inhibition of *FASN* expression by combining miR-27a-3p, and regulates TAG synthesis and fatty acid composition in BMECs [41]. Circ08409 relieves the inhibitory effect of miR-133a on *TGFB2* expression by binding it and subsequently modulating cell proliferation and apoptosis in BMECs [42]. In the current study, some ncRNAs and key genes in the HPM axis were identified under HS in the ceRNA regulatory network, such as novel_circ_000126-bta-miR-211- *HSPH1* and novel_circ_002325- bta-miR-218/bta-miR-33a- *GHRHR* in the hypothalamus. In the pituitary, the mainly ceRNA network was found in novel_circ_013439- miR-9-z- *PRL*, novel_circ_006323- bta-miR-124a- *GH1*, and novel_circ_020252- miR-7864-z -*IGF1*. Moreover, the subnetworks of novel_circ_011229- bta-miR-335- *PRLR* and novel_circ_016821- bta-miR-129- *IGF1* were established in mammary gland. The expression of miR-23a, miR-27a, miR-27b, miR-103 and miR-200a can be upregulated by *PRL* [43]. One study has shown that miR-15a decreases BMEC viability and lactation, and regulates *GHR* expression [44]. Additionally, miR-29a-3p inhibits cell proliferation, *PRL* and *GH* expression of prolactinoma cells, and promotes apoptosis by inhibiting *IGF-1* [45]. Therefore, we hypothesized that dairy cows may respond to the thermal environment by regulating the expression of various RNAs and key pathways in the HPM axis, affecting hormone secretion and lactation performance. The specific mechanisms should be investigated in future studies.

In conclusion, this study indicates that HS may affect the stress response and lactation physiology of dairy cows by regulating the secretion of endocrine-related hormones and release of neurotransmitters, and the expression of circRNAs, miRNAs, and mRNAs in HPM axis-related tissues. The differential gene expression is closely related to the MAPK signaling pathway, which may be a potential regulatory pathway. Moreover, the established ceRNA (circRNA-miRNA-mRNA) regulatory networks provide a theoretical basis for the molecular mechanism of the stress response and lactation regulation of ncRNAs in HPM axis-related tissues under HS.

## 4. Materials and Methods

### 4.1. Animals and Sample Collection

The temperature and humidity index (THI) of the cowshed, and the rectal temperature, and respiratory rate of Holstein cows were continuously monitored under the same management level in summer (August, heat stress group (HS), *n* = 20) and winter (December, Non heat stress group (NHS), *n* = 20) in Huai’an Jielong Animal Husbandry Co., Ltd. (Huai’an, China). Information about parity, lactation days and milk yield of the cows in NHS group and HS group are shown in Table 1. There was no significant difference in parity and lactation days between NHS and HS groups (*p* > 0.05), but the milk yield decreased significantly in HS groups (*p* < 0.05).

The data of THI, rectal temperature, and respiratory rate are shown in Table 2. The temperature (T, °C) and relative humidity (RH, %) of the cowshed were measured daily at 10:00 and 20:00 for a month. Using electronic thermometers at six fixed points in the cowshed. THI criteria were derived from the NRC (1971), and the THI was calculated according to the following equation: THI = (1.8 × T + 32) − (0.55 − 0.55 × RH) × (1.8 × T − 26), where T is the temperature (°C) and RH is the relative humidity (%) [46]. Rectal temperature and respiratory rate were measured using an animal thermometer and stopwatch, respectively, at 10:00 and 20:00 daily during the experiment.

Blood samples (10 mL) from the NHS and HS groups of Holstein cows were obtained from the caudal vein before morning feedings during the trial period and collected into tubes until coagulation. The collected samples were centrifuged at 3000 rpm for 10 min at 4 °C and stored at −20 °C for subsequent serum endocrine hormone and antioxidant index measurements. Blood sample collection was approved and implemented by Huai’an Jielong Animal Husbandry Co., Ltd. (Huai’an, China).

Six lactating Holstein cows (NHS: *n* = 3; HS: *n* = 3) were randomly selected from the NHS and HS groups. Cows were purchased and slaughtered, and the hypothalamus (HS: HS_H1, HS_H2, and HS_H3; NHS: NHS_H1, NHS_H2, and NHS_H3), pituitary (HS: HS_P1, HS_P2, and HS_P3; NHS: NHS_P1, NHS_P2, and NHS_P3), and mammary gland tissues (HS: HS_M1, HS_M2, and HS_M3; NHS: NHS_M1, NHS_M2, and NHS_M3) were collected and frozen in liquid nitrogen within 20 min after death for subsequent experiments. Sample collection information is shown in Table 3.

### 4.2. Detection of Endocrine Hormones, Antioxidant Enzyme Activity, and Heat Shock Proteins

Serum levels of endocrine hormones, corticotropin releasing hormone (CRH), thyrotropin releasing hormone (TRH), prolactin releasing factor (PRF), prolactin release inhibitor (PIF), adrenocorticotropic hormone (ACTH), theroid stimulating hormone (TSH), prolactin (PRL), cortisol (COR), thyroid hormone (T4), triiodothyronine (T3), growth hormone (GH), insulin (INS), and insulin-like factor 1 (IGF-1), and heat shock protein 70 (HSP70) and heat shock protein 90 (HSP90) were determined using an ELISA Assay Kit (Meimian, Jiangsu Feiya Biological Technology Co. Ltd.,Yancheng, China) according to the manufacturer’s protocol. The absorbance was read at 450 nm using a microplate reader. Hormonal content was calculated based on absorbance. Commercial kits were used to measure concentrations of malondialdehyde (MDA), superoxide dismutase (SOD), glutathione peroxidase (GSH-Px) and lactate dehydrogenase (LDH) in the serum according to the manufacturer’s instructions (Jiancheng Biological Project, Nanjing, China). The activity of these antioxidant enzymes was calculated based on absorbance.

### 4.3. Histological Evaluation

To prepare slides for histological evaluation, fresh hypothalamus, pituitary, and mammary gland samples were fixed in 4% paraformaldehyde and tissue sections were subsequently dehydrated through steps of graded alcohol, cleared in xylene, and embedded in paraffin blocks. Sections of 6 μm thickness were cut from each block. The deparaffinized sections were stained with hematoxylin and eosin. The tissue sections were then observed using a microscope (Olympus Corporation, Tokyo, Japan).

### 4.4. Detection of Neurotransmitters

High performance liquid chromatography- tandem mass spectrometry (HPLC-MS/MS) was used to detect γ-Aminobutyric acid (GABA), acetylcholine (ACh), dopamine (DA), and norepinephrine (NE) content. β-Endorphin (β-EP) ELISA Assay Kit (Nanjing Ruiyuan Biotechnology Co., Ltd., Nanjing, China) was used to determine it according to the manufacturer’s protocol. The absorbance was read at 450 nm using a microplate reader and β-EP content was calculated based on absorbance.

### 4.5. RNA Extraction and Sequencing

RNA-seq was performed using hypothalamus, pituitary, and mammary gland tissues sample. First, total RNA was extracted from tissue samples. Ribosomal RNAs (rRNAs) were depleted to construct a total RNA-seq library. RNAs were fragmented into short fragments using fragmentation buffer and reverse-transcribed into cDNA with random primers. Second-strand cDNA was synthesized by DNA polymerase I, RNase H, dNTP (dUTP instead of dTTP) and buffer. The cDNA fragments were purified using the QiaQuick PCR extraction kit (Qiagen, Venlo, The Netherlands), end-repaired, poly(A) added, and ligated to Illumina sequencing adapters. Uracil-N-Glycosylase (UNG) was used to digest the second-strand cDNA. The digested products were size-selected using agarose gel electrophoresis, amplified by PCR, and sequenced using Illumina Nova-Seq 6000 by Gene Denovo Biotechnology Co., Ltd. (Guangzhou, China).

After total RNA was extracted, Small RNA molecules in a size range of 18–30 nt were enriched using polyacrylamide gel electrophoresis (PAGE). Then, the 3′-adapters were added and the 36–44 nt RNAs were enriched. The 5′-adapters were then ligated to the RNAs and the ligation products were reverse-transcribed using PCR amplification. The 140–160 bp size PCR products were enriched to generate a small RNA library and sequenced using Illumina Nova-Seq 6000.

### 4.6. Identification of circRNAs, miRNAs and mRNAs

To obtain high quality clean reads, raw reads were filtered using fastp (v0.18.0). Short reads alignment tool Bowtie2 (v.2.2.8) was used for mapping reads to ribosome RNA (rRNA) database. The rRNA removed reads from each sample were then mapped to the cow reference genome by HISAT2 (v2.1.1). After alignment with reference genome, the reads that could be mapped to the genomes were discarded, and the unmapped reads were collected for circRNA identification. From both ends of the unmapped reads, 20 mers were extracted and aligned to the reference genome to identify unique anchor positions within the splice site. Anchor reads that aligned in the reversed orientation (head-to tail) indicated circRNA splicing and were subjected to find_circ to identify circRNAs. The identified circRNAs were subjected to statistical analyses for type, and chromosome and length distribution.

All clean tags were searched against the miRBase database to identify known miRNAs. The unannotated tags were aligned with the cow reference genome according to their genome positions and hairpin structures predicted by the Mireap_v0.2 software to identify novel miRNA candidates.

Transcript reconstruction was carried out with the Stringtie software (v1.3.4), which, together with HISAT2, allow biologists to identify new genes and new splice variants of known genes.

### 4.7. Differentially Expressed of circRNAs, miRNAs and mRNAs

To identify DEcircRNAs, DEmiRNAs and DEmRNAs in the HPM axis tissues between the NHS and HS groups, the edgeR package (http://www.rproject.org/ (accessed on 15 September 2021)) was used. We identified circRNAs and miRNAs with fold change (FC) ≥ 2 and *p*-value < 0.05 in a comparison between groups as significantly DEcircRNAs and DEmiRNAs, respectively. Significantly DEmRNAs were identified with a FC ≥ 2 and false discovery rate (FDR) < 0.05.

### 4.8. Function Enrichment Analysis

To understand the potential roles of DEcircRNAs, DEmiRNAs and DEmRNAs between NHS and HS groups, Gene ontology (GO) enrichment analysis and the Kyoto encyclopedia of genes and genomes (KEGG) pathway analyses were performed to investigate their biological function using DAVID (http://david.abcc.ncifcrf.gov/ (accessed on 24 September 2021)).

### 4.9. Construction of the Competing Endogenous RNA (DEcircRNA-DEmiRNA-DEmRNA) Regulatory Network

The ceRNA network was constructed based on the ceRNA theory as follows: (1) The expression correlation between mRNA-miRNA or circRNA-miRNA was evaluated using the Spearman Rank correlation coefficient (SCC). Pairs with SCC < −0.7 were selected as negatively coexpressed circRNA-miRNA pairs or mRNA-miRNA pairs, and both mRNA and circRNA were miRNA target genes, and all RNAs were differentially expressed. (2) The expression correlation between circRNA-mRNA was evaluated using the Pearson correlation coefficient (PCC). Pairs with PCC > 0.9 were selected as coexpressed circRNA-mRNA pairs, and both mRNA and circRNA in these pairs were targeted and negatively coexpressed with a common miRNA. (3) A hypergeometric cumulative distribution function test was used to test whether the common miRNA sponges between the two genes were significant. As a result, only the gene pairs with a *p*-value less than 0.05 were selected.

### 4.10. Quantitative Real-Time Polymerase Chain Reaction (RT-qPCR) Validation

Total RNA was extracted using TRIzol Reagent (Invitrogen, Carlsbad, CA, USA) and the density of sample RNA was quantified spectrophoto-metrically at 260/280 nm. The RNA was reverse-transcribed using Prime Script^TM^ RT Master Mix (TaKaRa, Otsu, Japan) according to the manufacturer’s protocol. The sequences of the primers are listed in (Supplementary Tables S7 and S8). All RT-qPCR runs were performed on a StepOne Plus Real-Time PCR System (Applied Biosystems, Foster City, CA, USA) using the AceQ qPCR SYBR Green Master Mix (Vazyme Biotech Co., Ltd., Nanjing, China) in a reaction volume of 20 µL. The relative quantification of miRNAs, circRNAs, and mRNAs were normalized to that of U6 or β-actin using the 2^−ΔΔCt^ method.

### 4.11. Statistical Analysis

The statistical analyses were performed in SPSS version 20.0 (SPSS Inc., Chicago, IL, USA) and the graphs were generated using GraphPad Prism 8.4.2 (La Jolla, CA, USA). The significance of differences in physiological and biochemical parameters were determined using One-way ANOVA with Duncan’s multiple range test, and the data are expressed as mean and standard error of mean (SEM). *p*-values < 0.05 were considered significant.

## Figures and Tables

**Table 1:** Characteristics of the Holstein dairy cows used in the study.

Items	NHS (*n* = 20)	HS (*n* = 20)	SEM	*p*-Value
Parity	1.50	1.35	0.67	0.49
Lactation day(s)	190.60	188.05	17.19	0.65
Milk yield(kg/d)	38.01 ^a^	33.56 ^b^	5.74	<0.05

NHS, Non heat stress group; HS, heat stress group. The data are expressed as means, different superscript letters in the same row indicate significant differences between groups (*p* < 0.05).

**Table 2:** Environmental temperature and humidity index (THI), and rectal temperature and respiratory rate of Holstein dairy cows.

Groups	Temperature and Humidity Index (THI)	Average Rectal Temperature (℃, *n* = 20)	Average Respiratory Rate (times/min, *n* = 20)
NHS	58.44 ± 2.09	38.41 ± 0.82	40.94 ± 2.75
HS	83.98 ± 2.71 ***	39.68 ± 0.24	69.13 ± 2.44 ***

NHS, non heat stress group; HS, heat stress group. Data are presented as the mean ± SEM; *** *p* < 0.001.

**Table 3:** Sample collection information.

Groups	Hypothalamus	Pituitary	Mammary Gland
NHS (*n* = 3)	NHS_H1	NHS_P1	NHS_M1
	NHS_H2	NHS_P2	NHS_M2
	NHS_H3	NHS_P3	NHS_M3
HS (*n* = 3)	HS_H1	HS_P1	HS_M1
	HS_H2	HS_P2	HS_M2
	HS_H3	HS_P3	HS_M3

NHS, non heat stress group; HS, heat stress group.

## Data Availability

All raw transcriptome data reported in current article have been deposited to the National Center for Biotechnology Information (https://www.ncbi.nlm.nih.gov/sra/PRJNA885909 (accessed on 30 September 2022)).

## Supplementary Materials

The following supporting information can be downloaded at: https://www.mdpi.com/article/10.3390/ijms24010888/s1.

## References

[1] Becker C.A., Collier R.J., Stone A.E. (2020). Invited review: Physiological and behavioral effects of heat stress in dairy cows. J. Dairy Sci..

[2] Chen K.L., Wang H.L., Jiang L.Z., Qian Y., Yang C.X., Chang W.W., Zhong J.F., Xing G.D. (2020). Heat stress induces apoptosis through disruption of dynamic mitochondrial networks in dairy cow mammary epithelial cells. Vitr. Cell. Dev. Biol. Anim..

[3] Wohlgemuth S.E., Ramirez-Lee Y., Tao S., Monteiro A.P.A., Ahmed B.M., Dahl G.E. (2016). Short communication: Effect of heat stress on markers of autophagy in the mammary gland during the dry period. J. Dairy Sci..

[4] Tao S., Orellana R.M., Weng X., Marins T.N., Dahl G.E., Bernard J.K. (2018). Symposium review: The influences of heat stress on bovine mammary gland function. J. Dairy Sci..

[5] Han J., Shao J., Chen Q., Sun H., Guan L., Li Y., Liu J., Liu H. (2019). Transcriptional changes in the hypothalamus, pituitary, and mammary gland underlying decreased lactation performance in mice under heat stress. FASEB J..

[6] Cakir I., Nillni E.A. (2019). Endoplasmic Reticulum Stress, the Hypothalamus, and Energy Balance. Trends Endocrinol. Metab..

[7] Duan B., Xu X.Z.S. (2019). How to Break a Fever: A Feedback Circuit for Body Temperature Control. Neuron.

[8] Ni Y., Chen Q., Cai J., Xiao L., Zhang J. (2021). Three lactation-related hormones: Regulation of hypothalamus-pituitary axis and function on lactation. Mol. Cell Endocrinol..

[9] Hamada M., Nishigawa T., Maesono S., Aso K., Ikeda H., Furuse M. (2019). Decreased stress-induced depression-like behavior in lactating rats is associated with changes in the hypothalamic-pituitary-adrenal axis, brain monoamines, and brain amino acid metabolism. Stress.

[10] Weitzel J.M., Viergutz T., Albrecht D., Bruckmaier R., Schmicke M., Tuchscherer A., Koch F., Kuhla B. (2017). Hepatic thyroid signaling of heat-stressed late pregnant and early lactating cows. J. Endocrinol..

[11] Ni Y., Wu F., Chen Q., Cai J., Hu J., Shen J., Zhang J. (2020). Long noncoding RNA and mRNA profiling of hypothalamic-pituitary-mammary gland axis in lactating sows under heat stress. Genomics.

[12] Wang Y., Fang J., Zeng H.F., Zhong J.F., Li H.X., Chen K.L. (2022). Identification and bioinformatics analysis of differentially expressed milk exosomal microRNAs in milk exosomes of heat-stressed Holstein cows. Funct. Integr. Genom..

[13] Okholm T.L.H., Sathe S., Park S.S., Kamstrup A.B., Rasmussen A.M., Shankar A., Chua Z.M., Fristrup N., Nielsen M.M., Vang S. (2020). Transcriptome-wide profiles of circular RNA and RNA-binding protein interactions reveal effects on circular RNA biogenesis and cancer pathway expression. Genome Med..

[14] Wang D., Chen Z., Zhuang X., Luo J., Chen T., Xi Q., Zhang Y., Sun J. (2020). Identification of circRNA-Associated-ceRNA Networks Involved in Milk Fat Metabolism under Heat Stress. Int. J. Mol. Sci..

[15] Zhang M., Ma L., Liu Y., He Y., Li G., An X., Cao B. (2020). CircRNA-006258 Sponge-Adsorbs miR-574-5p to Regulate Cell Growth and Milk Synthesis via EVI5L in Goat Mammary Epithelial Cells. Genes.

[16] Thomson D.W., Dinger M.E. (2016). Endogenous microRNA sponges: Evidence and controversy. Nat. Rev. Genet..

[17] Huang G., Liang M., Liu H., Huang J., Li P., Wang C., Zhang Y., Lin Y., Jiang X. (2020). CircRNA hsa_circRNA_104348 promotes hepatocellular carcinoma progression through modulating miR-187-3p/RTKN2 axis and activating Wnt/beta-catenin pathway. Cell Death Dis..

[18] Li H., Xu J.D., Fang X.H., Zhu J.N., Yang J., Pan R., Yuan S.J., Zeng N., Yang Z.Z., Yang H. (2020). Circular RNA circRNA_000203 aggravates cardiac hypertrophy via suppressing miR-26b-5p and miR-140-3p binding to Gata4. Cardiovasc. Res..

[19] Sinha K., Das J., Pal P.B., Sil P.C. (2013). Oxidative stress: The mitochondria-dependent and mitochondria-independent pathways of apoptosis. Arch. Toxicol..

[20] Zeng H.F., Xu J., Wang X.L., Li S.J., Han Z.Y. (2022). Nicotinamide mononucleotide alleviates heat stress-induced oxidative stress and apoptosis in BMECs through reducing mitochondrial damage and endoplasmic reticulum stress. Ecotoxicol. Environ. Saf..

[21] Bagath M., Krishnan G., Devaraj C., Rashamol V.P., Pragna P., Lees A.M., Sejian V. (2019). The impact of heat stress on the immune system in dairy cattle: A review. Res. Vet. Sci..

[22] Helmreich D.L., Parfitt D.B., Lu X.Y., Akil H., Watson S.J. (2005). Relation between the hypothalamic-pituitary-thyroid (HPT) axis and the hypothalamic-pituitary-adrenal (HPA) axis during repeated stress. Neuroendocrinology.

[23] Babic Leko M., Gunjaca I., Pleic N., Zemunik T. (2021). Environmental Factors Affecting Thyroid-Stimulating Hormone and Thyroid Hormone Levels. Int. J. Mol. Sci..

[24] Pirahanchi Y., Toro F., Jialal I. (2022). Physiology, Thyroid Stimulating Hormone. StatPearls.

[25] Ben-Jonathan N., Hnasko R. (2001). Dopamine as a prolactin (PRL) inhibitor. Endocr. Rev..

[26] Yip S.H., Romano N., Gustafson P., Hodson D.J., Williams E.J., Kokay I.C., Martin A.O., Mollard P., Grattan D.R., Bunn S.J. (2019). Elevated Prolactin during Pregnancy Drives a Phenotypic Switch in Mouse Hypothalamic Dopaminergic Neurons. Cell Rep..

[27] Verma A., Sumi S., Seervi M. (2021). Heat shock proteins-driven stress granule dynamics: Yet another avenue for cell survival. Apoptosis.

[28] Saibil H. (2013). Chaperone machines for protein folding, unfolding and disaggregation. Nat. Rev. Mol. Cell Biol..

[29] Lanneau D., Brunet M., Frisan E., Solary E., Fontenay M., Garrido C. (2008). Heat shock proteins: Essential proteins for apoptosis regulation. J. Cell Mol. Med..

[30] Arya R., Mallik M., Lakhotia S.C. (2007). Heat shock genes—Integrating cell survival and death. J. Biosci..

[31] Pawlowski K., Lago-Novais D., Bevilacqua C., Mobuchon L., Crapart N., Faulconnier Y., Boby C., Carvalho G., Martin P., Leroux C. (2020). Different miRNA contents between mammary epithelial cells and milk fat globules: A random or a targeted process?. Mol. Biol. Rep..

[32] Zhang M., Cao M., Kong L., Liu J., Wang Y., Song C., Chen X., Lai M., Fang X., Chen H. (2020). MiR-204-5p promotes lipid synthesis in mammary epithelial cells by targeting SIRT1. Biochem. Biophys. Res. Commun..

[33] Chen Y., Li S., Zhang Y., Wang M., Li X., Liu S., Xu D., Bao Y., Jia P., Wu N. (2021). The lncRNA Malat1 regulates microvascular function after myocardial infarction in mice via miR-26b-5p/Mfn1 axis-mediated mitochondrial dynamics. Redox Biol..

[34] Sun Y., Liu W.Z., Liu T., Feng X., Yang N., Zhou H.F. (2015). Signaling pathway of MAPK/ERK in cell proliferation, differentiation, migration, senescence and apoptosis. J. Recept. Signal Transduct Res..

[35] Szyller J., Bil-Lula I. (2021). Heat Shock Proteins in Oxidative Stress and Ischemia/Reperfusion Injury and Benefits from Physical Exercises: A Review to the Current Knowledge. Oxid. Med. Cell. Longev..

[36] Li H., Liu Y., Gu Z., Li L., Liu Y., Wang L., Su L. (2018). p38 MAPK-MK2 pathway regulates the heat-stress-induced accumulation of reactive oxygen species that mediates apoptotic cell death in glial cells. Oncol. Lett..

[37] Zou L., Cheng G., Xu C., Liu H., Wang Y., Li N., Zhu C., Xia W. (2021). The role of miR-128-3p through MAPK14 activation in the apoptosis of GC2 spermatocyte cell line following heat stress. Andrology.

[38] Gupta A., Chauhan N.R., Chowdhury D., Singh A., Meena R.C., Chakrabarti A., Singh S.B. (2017). Heat stress modulated gastrointestinal barrier dysfunction: Role of tight junctions and heat shock proteins. Scand. J. Gastroenterol..

[39] Yong Y., Li J., Gong D., Yu T., Wu L., Hu C., Liu X., Yu Z., Ma X., Gooneratne R. (2021). ERK1/2 mitogen-activated protein kinase mediates downregulation of intestinal tight junction proteins in heat stress-induced IBD model in pig. J. Therm. Biol..

[40] Sen R., Ghosal S., Das S., Balti S., Chakrabarti J. (2014). Competing endogenous RNA: The key to posttranscriptional regulation. Sci. World J..

[41] Chen Z., Zhou J., Wang M., Liu J., Zhang L., Loor J.J., Liang Y., Wu H., Yang Z. (2020). Circ09863 Regulates Unsaturated Fatty Acid Metabolism by Adsorbing miR-27a-3p in Bovine Mammary Epithelial Cells. J. Agric. Food Chem..

[42] Chen Z., Liang Y., Lu Q., Nazar M., Mao Y., Aboragah A., Yang Z., Loor J.J. (2021). Cadmium promotes apoptosis and inflammation via the circ08409/miR-133a/TGFB2 axis in bovine mammary epithelial cells and mouse mammary gland. Ecotoxicol. Environ. Saf..

[43] Dysin A.P., Barkova O.Y., Pozovnikova M.V. (2021). The Role of microRNAs in the Mammary Gland Development, Health, and Function of Cattle, Goats, and Sheep. Noncoding RNA.

[44] Li H.M., Wang C.M., Li Q.Z., Gao X.J. (2012). MiR-15a decreases bovine mammary epithelial cell viability and lactation and regulates growth hormone receptor expression. Molecules.

[45] Xia J., Li S., Ma D., Guo W., Long H., Yin W. (2021). MicroRNA293p regulates the betacatenin pathway by targeting IGF1 to inhibit the proliferation of prolactinoma cells. Mol. Med. Rep..

[46] Pinto S., Hoffmann G., Ammon C., Amon T. (2020). Critical THI thresholds based on the physiological parameters of lactating dairy cows. J. Therm. Biol..

