# Supplementary material for: Comprehensive Profiling of ceRNA (circRNA-miRNA-mRNA) Networks in Hypothalamic-Pituitary-Mammary Gland Axis of Dairy Cows under Heat Stress
Source: Int J Mol Sci. 2023 Jan 3;24(1):888. doi: 10.3390/ijms24010888 (PMC9821774; doi:10.3390/ijms24010888)
Supplement: Supplementary file 1 [file ijms-24-00888-s001.zip › Supplementary Figure annotation.pdf]

Figure S1. GO functional enrichment analysis on the taget genes of DE miRNAs in the hypothalamus, pituitary, mammary gland in dairy cows under heat stress.

Figure S2. GO functional enrichment analysis on DE mRNAs in the hypothalamus, pituitary, mammary gland in dairy cows under heat stress.
