# Supplementary figures and images for: Comprehensive Profiling of ceRNA (circRNA-miRNA-mRNA) Networks in Hypothalamic-Pituitary-Mammary Gland Axis of Dairy Cows under Heat Stress
Source: Int J Mol Sci. 2023 Jan 3;24(1):888. doi: 10.3390/ijms24010888 (PMC9821774; doi:10.3390/ijms24010888)

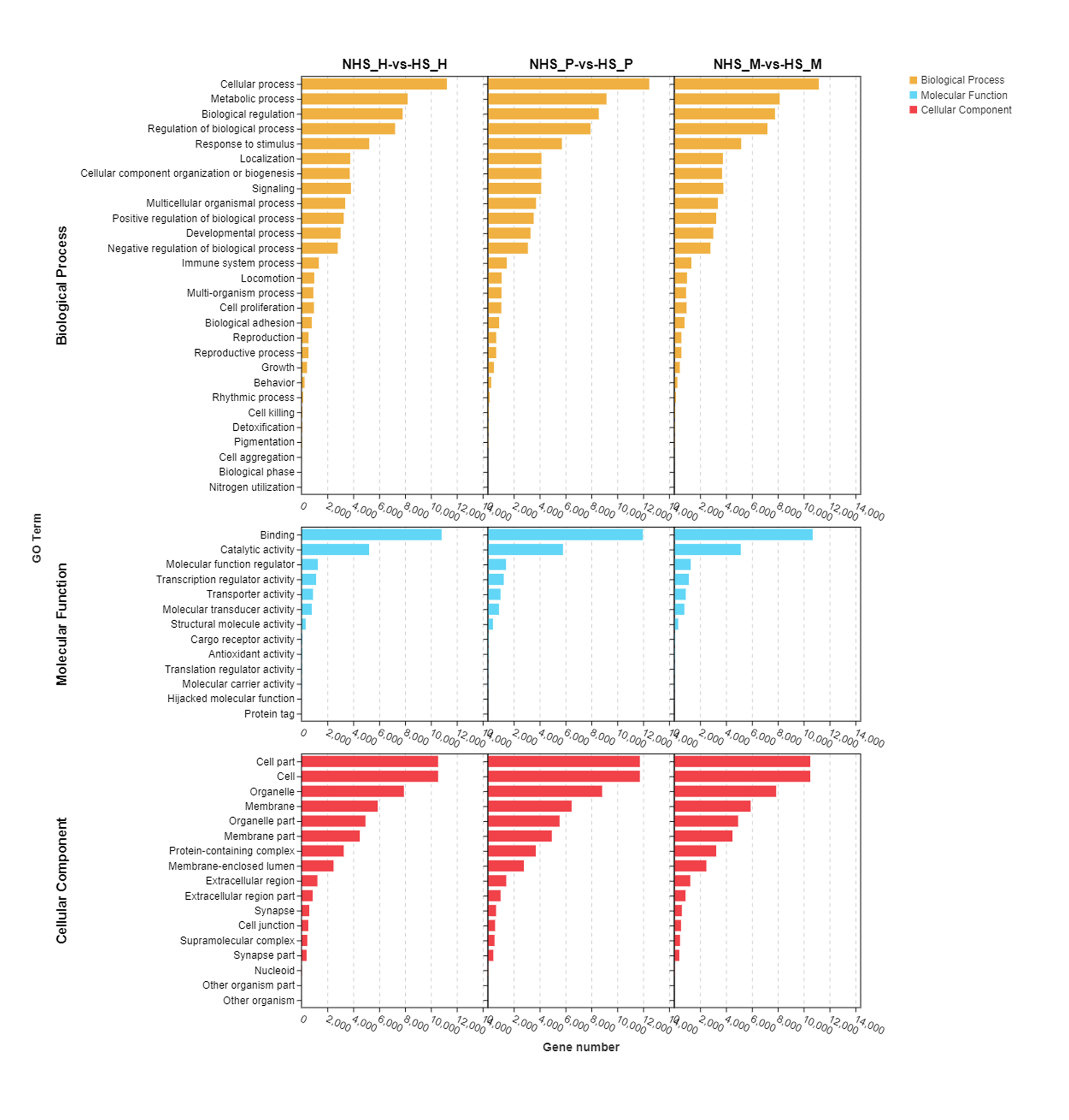

Supplement: Supplementary file 1 [file ijms-24-00888-s001.zip › Supplementary FigureS1.tif]

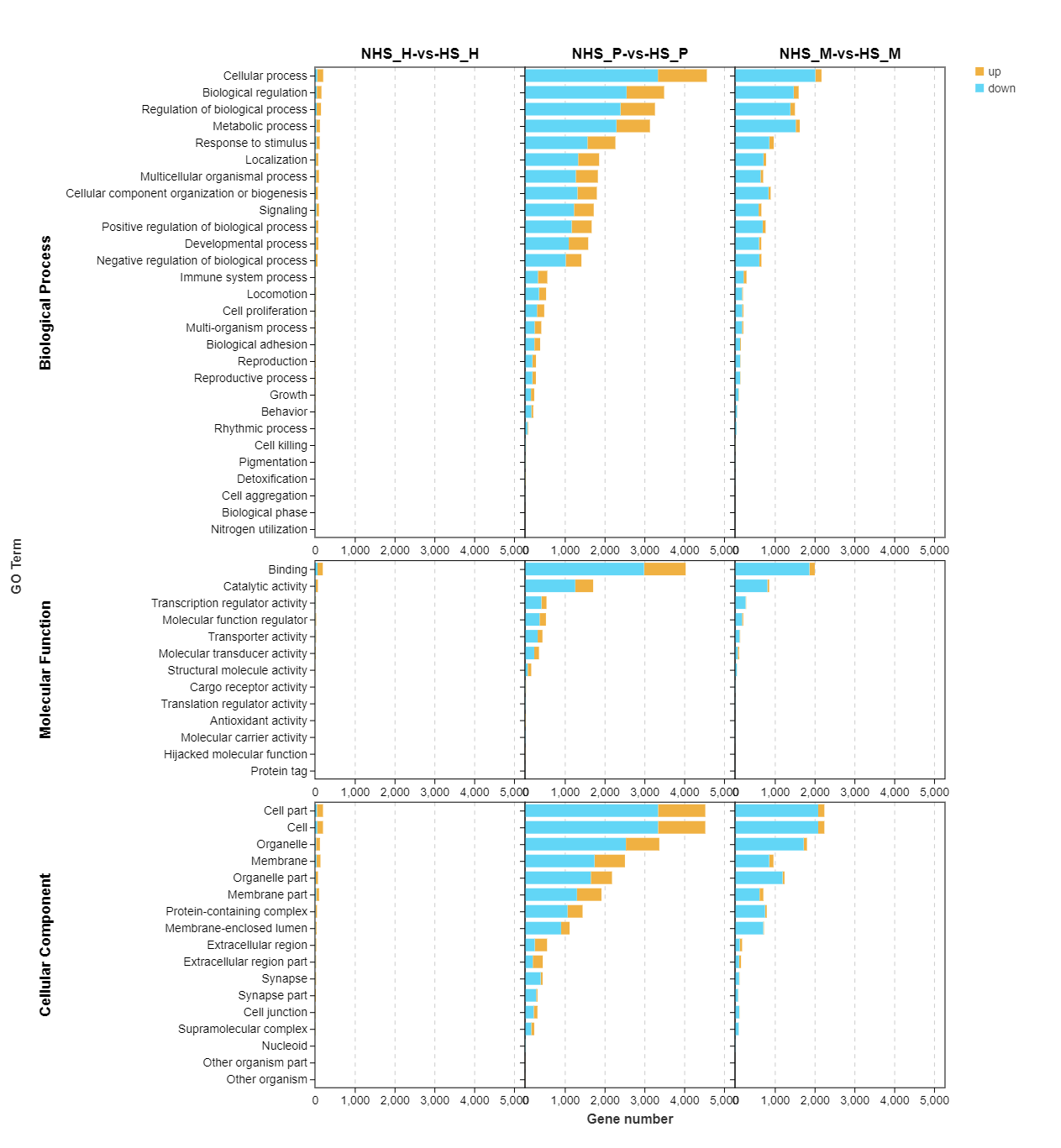

Supplement: Supplementary file 1 [file ijms-24-00888-s001.zip › Supplementary FigureS2.png]
